# Tyrosine dephosphorylation enhances the therapeutic target activity of epidermal growth factor receptor (EGFR) by disrupting its interaction with estrogen receptor (ER)

**DOI:** 10.18632/oncotarget.3645

**Published:** 2015-04-10

**Authors:** Shao Ma, Ning Yin, Xiaomei Qi, Sandra L. Pfister, Mei-Jie Zhang, Rong Ma, Guan Chen

**Affiliations:** ^1^ Department of Pharmacology and Toxicology, Medical College of Wisconsin, Milwaukee, WI 53226, USA; ^2^ Department of Breast Surgery, QiLu Hospital of Shandong University, Jinan, Shandong Province 250012, China; ^3^ Division of Biostatistics, Medical College of Wisconsin, Milwaukee, WI 53226, USA; ^4^ Zablocki Veterans Affairs Medical Center, Milwaukee, WI 53226, USA

**Keywords:** protein tyrosine phosphatase H1 (PTPH1), EGFR, ER, protein-protein-interactions, therapeutic target activity

## Abstract

Protein-protein interactions can increase or decrease its therapeutic target activity and the determining factors involved, however, are largely unknown. Here, we report that tyrosine-dephosphorylation of epidermal growth factor receptor (EGFR) increases its therapeutic target activity by disrupting its interaction with estrogen receptor (ER). Protein tyrosine phosphatase H1 (PTPH1) dephosphorylates the tyrosine kinase EGFR, disrupts its interaction with the nuclear receptor ER, and increases breast cancer sensitivity to small molecule tyrosine kinase inhibitors (TKIs). These effects require PTPH1 catalytic activity and its interaction with EGFR, suggesting that the phosphatase may increase the sensitivity by dephosphorylating EGFR leading to its dissociation with ER. Consistent with this notion, a nuclear-localization defective ER has a higher EGFR-binding activity and confers the resistance to TKI-induced growth inhibition. Additional analysis show that PTPH1 stabilizes EGFR, stimulates the membranous EGFR accumulation, and enhances the growth-inhibitory activity of a combination therapy of TKIs with an anti-estrogen. Since EGFR and ER both are substrates for PTPH1 *in vitro* and in intact cells, these results indicate that an inhibitory EGFR-ER protein complex can be switched off through a competitive enzyme-substrate binding. Our results would have important implications for the treatment of breast cancer with targeted therapeutics.

## INTRODUCTION

The epidermal growth factor receptor (EGFR) belongs to the plasma membrane tyrosine kinase family and plays a critical role in cell growth and malignant development [1, 2]. Upon binding by its ligand EGF, EGFR is dimerized and activated by auto-phosphorylation on tyrosine residues leading to activation of downstream proliferative pathways such as Ras/MAPKs (mitogen-activated protein kinases) [2]. This results in increased cell proliferation and malignant progression [2]. EGFR is overexpressed in breast cancer and is one of the first identified molecular targets for therapeutic intervention [1, 3]. EGFR can be inhibited by blocking the extracellular ligand binding domain with an anti-EGFR antibody or by suppressing its phosphorylation with a small molecular tyrosine kinase inhibitor (TKI) via binding to the ATP-binding pocket of its cytoplasmic tyrosine kinase domain [4]. Although preclinical studies showed promising anti-tumor activity of TKIs in breast cancer, results from clinical trials are disappointing [3]. Moreover, TKIs suppress the malignant growth by inhibiting EGFR tyrosine phosphorylation and effects of a protein tyrosine phosphatase on its therapeutic activity however have not been reported [5]. This could be a key mechanism to increase the therapeutic target activity of EGFR.

Estrogen receptor α (ERα or ER) is expressed in about 70% of breast cancer. Activation of ER by estrogens leads to increased expression of ER target genes important for breast cancer growth [6]. ER is the only therapeutic target for anti-estrogens such as tamoxifen (TAM) [7]. However, about 50% of ER positive (ER+) breast cancer are refractory to the hormone therapy and there is an urgent need to develop novel strategies to overcome the resistance [7]. Increased EGFR expression is associated with decreased sensitivity to anti-estrogens [8] and EGFR-forced expression in ER+ breast cancer further induces hormone-independent growth [9]. Moreover, ER binds EGFR and this interaction is enhanced in TAM-resistant breast cancer [10, 11], indicating an inhibitory activity of this protein-complex in ER therapeutic target activity. Mechanisms dictating this unique nuclear-membrane receptor interaction, however, remain unknown. In addition, it is unknown whether the EGFR-ER interaction impacts the therapeutic target activity of EGFR. Since the ER-EGFR signal cross-talk is bidirectional [12], the complex formation of EGFR with ER may also play an important role in breast cancer sensitivity to TKIs.

Protein-tyrosine phosphatase H1 (PTPH1, the gene name: *PTPN3*) is a 120-kDa protein that belongs to the non-transmembrane PTP super-family [13, 14]. Previous genetic analysis showed that PTPH1 is mutated in human colon cancer but the functional consequence of this mutation remains unknown [15]. Recent studies showed that PTPH1 cooperates with p38γ MAPK to promote Ras oncogenesis through overexpression [16–18] and PTPH1 mutations further increase its oncogenic activity [19]. Importantly, PTPH1 is overexpressed in breast cancer and promotes breast cancer growth through increasing vitamin D receptor (VDR) cytoplasmic accumulation [20]. Our recent studies further demonstrated that PTPH1 dephosphorylates ER at Y537, increases ER stability and nuclear accumulation, and enhances breast cancer sensitivity to anti-estrogens [21]. In this report, we tested the hypothesis that PTPH1 may decrease EGFR tyrosine phosphorylation thereby regulating the ER-EGFR interaction and breast cancer sensitivity to TKIs. Our results showed that PTPH1 disrupts the ER-EGFR complex through catalyzing EGFR tyrosine dephosphorylation leading to increased breast cancer sensitivities to TKIs. These results, together with the sensitizing effect of PTPH1 to anti-estrogens [21], indicate that the EGFR-ER interaction is an intrinsic resistant factor to their targeted therapies and this inhibitory complex can be disrupted by PTPH1-induced dephosphorylation.

## RESULTS

### PTPH1 dephosphorylates EGFR/Y1173 in breast cancer cells

**Figure 1:**
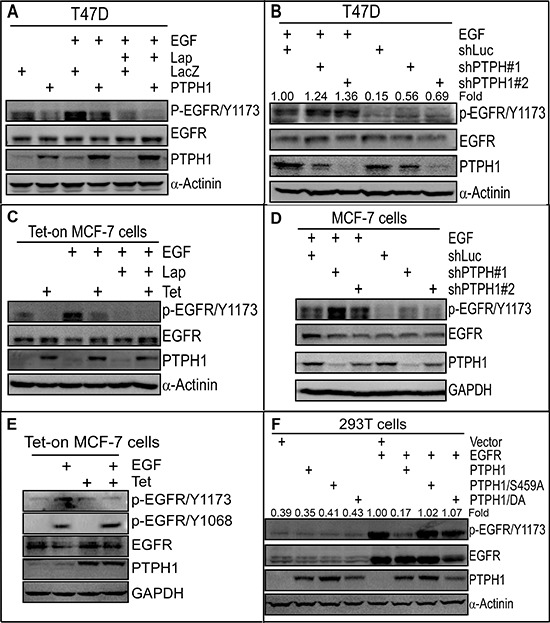
PTPH1 dephosphorylates EGFR at Y1173 in breast cancer cells **A.** PTPH1 expression decreases levels of p-EGFR/Y1173 in T47D cells. Indicated cells were treated with Lap (5 μM) or control vehicle for 5 min, followed by incubation with or without EGF (10 ng/ml) for another 5 min, and then analyzed by Western blot (WB). **B.** PTPH1 depletion increases levels of p-EGFR/Y1173 in T47D cells. PTPH1 stably depleted (or control shLuc) cells [20] were incubated with EGF as in **A** and assayed for protein expression and phosphorylation. The fold change was obtained by dividing p-EGFR/Y1173 bands with the corresponding EGFR and expressed as relative to shLuc + EGF. **C–E.** PTPH1 expression decreases p-EGFR/Y1173 levels without affecting p-EGFR/Y1068 in MCF-7 cells. Tet-on PTPH1 MCF-7 cells were treated with Lap and/or EGF as in **A** and analyzed by WB using indicated specific antibodies **(C–E)**. PTPH1 depletion increases levels of p-EGFR/Y1173 in MCF-7 cells. PTPH1 stably depleted cells were treated with EGF as in B and analyzed by WB **(D)**. **F.** PTPH1 dephosphorylates EGFR/Y1173 by co-transfection in 293T cells. Indicated constructs were co-transfected in 293T cells for 48 h and protein expression and phosphorylation were analyzed by direct WB. The fold change was obtained by dividing p-EGFR/Y1173 bands with the corresponding EGFR and expressed as relative to EGFR transfection. In all of these studies **(A–F)** similar results were obtained by at least two separate experiments.

Tyrosine phosphorylation is essential for EGFR to activate downstream mitogenic pathways [22] and acts as the foundation for targeted therapy with TKIs [23]. Our previous studies demonstrated that the tyrosine phosphatase PTPH1 dephosphorylates EGFR at Y1173 in cell-free system [17]. We wanted further to determine if PTPH1 decreases EGFR tyrosine phosphorylation in breast cancer cells. Results (Figure 1A) showed that stable PTPH1 expression in T47D breast cancer cells decreases levels of endogenous and EGF-induced tyrosine phosphorylation of EGFR at Y1173 (p-EGFR/Y1173). Moreover, knockdown of endogenous PTPH1 by two separate shRNAs increases p-EGFR/Y1173 levels with and without EGF treatment (Figure 1B). PTPH1 also negatively regulates p-EGFR/Y1173 levels in MCF-7 breast cancer cells (Figures 1C, 1D). Moreover, PTPH1 overexpression dephosphorylates EGFR at Y1173 but not at Y1068 (Figure 1E). These results together demonstrate that PTPH1 dephosphorylates EGFR/Y1173 in breast cancer cells.

We previously showed that p38γ MAPK phosphorylates PTPH1/S459, which is required for PTPH1 to increase Ras-dependent growth and to inhibit stress-induced cell death [17]. We next examined if S459 is required for the PTPH1 catalytic activity to dephosphorylate EGFR/Y1173 as compared with the positive control PTPH1/DA {a phosphatase-deficient trapping mutant [24]}. Transient PTPH1 expression significantly decreases levels of the co-expressed EGFR phosphorylation at Y1173, whereas neither of its mutants has such effect (Figure 1F). Together, these results demonstrate that PTPH1 efficiently catalyzes EGFR/Y1173 dephosphorylation, which may play a role in the therapeutic target activity of EGFR.

### PTPH1 increases breast cancer sensitivity to tyrosine kinase inhibitors (TKIs)

Small molecule TKIs inhibit cancer growth by suppressing EGFR tyrosine phosphorylation at multiple residues [2, 23]. It is not known, however, whether a protein tyrosine phosphatase can regulate the growth-inhibitory activity of these inhibitors by catalyzing EGFR tyrosine dephosphorylation. Since PTPH1 inhibits the EGFR/Y1173 phosphorylation, we next examined if it may regulate breast cancer sensitivity to TKIs. PTPH1 was overexpressed in MCF-7 cells by a tetracycline-inducible system (Tet-on) and in T47D cells by stable retroviral infection [20, 21]. To stably deplete endogenous PTPH1 proteins, cells were infected with lentivirus expressing shLuc (control) or shRNAs targeting two separate PTPH1 sequences, followed by antibiotic selection [20, 21]. Cells, with or without PTPH1 overexpression or depletion, were incubated with lapatinib (Lap), an EGFR/Her-2 dual inhibitor currently used in clinical trials [4]. Effects on breast cancer cell growth were assessed by colony formation assays [20, 21].

**Figure 2:**
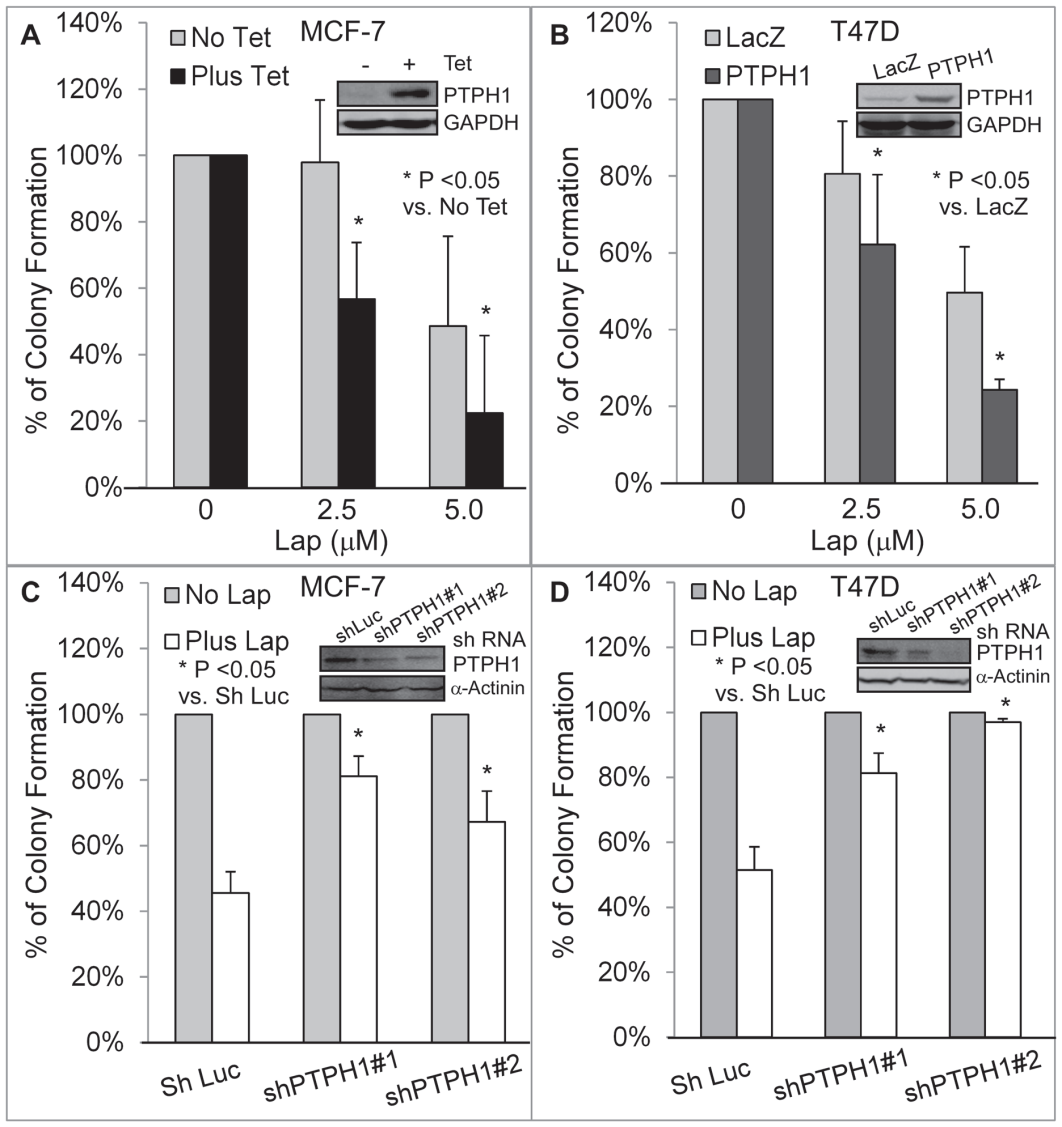
PTPH1 confers breast cancer cell sensitivity to lapatinib (Lap) **A, B.** PTPH1 overexpression increases the growth inhibition by Lap in MCF-7 **(A)** and T47D **B** cells. PTPH1 was overexpressed by a Tet-on system or a stable transfection and resultant cells were incubated with Lap or solvent for approximately 2 weeks for colony formation. Results shown are normalized colony numbers to respective solvent control (mean ± SD, *n* = 3) [21], with the inserts showing the ectopically expressed PTPH1 protein (48 hr after incubation with and without Tet for MCF-7 for **A**) **C, D.** PTPH1 silencing leads to the resistance to Lap-induced growth inhibition in MCF-7 **(C)** and T47D **(D)** cells. PTPH1 depleted cells were incubated with Lap (5.0 μM) or solvent and colony formation was assessed and analyzed as discussed above (mean ± SD, *n* = 3), with inserts showing a decreased PTPH1 expression by shPTPH1#1/2.

Results (Figures 2A–2D; Supplementary Figures 1A/1B) show that PTPH1 overexpression enhances the Lap-induced growth inhibition in both cell lines, whereas its depletion resulted in an opposite effect. A similar sensitizing effect of Tet-induced PTPH1 expression was also demonstrated in 231 breast cancer cells (Supplementary Figure 1C). However, PTPH1 expression has no effect on 17-alllylaminogeldanamycin {17-AAG, an inhibitor of heat shock protein 90 [25]}-induced growth inhibition (Supplementary Figure 1D). In addition, PTPH1 also increases breast cancer sensitivity to another TKI gefitinib (Gef), a specific EGFR inhibitor that has also been used clinically [26] (Supplementary Figures 2A–2E). These results together demonstrate that PTPH1 is a novel determinant of breast cancer sensitivity to EGFR-targeted therapies with TKIs.

### PTPH1 confers the breast cancer sensitivity by disrupting the EGFR-ER interaction

**Figure 3:**
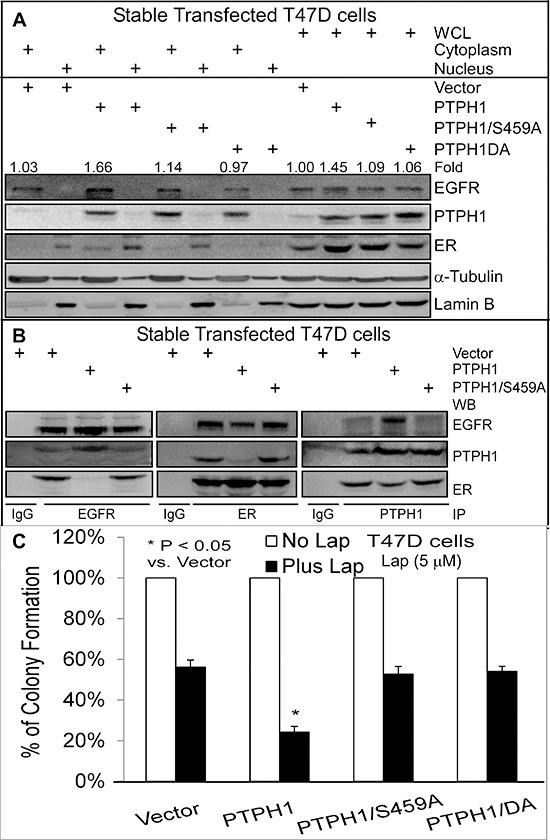
PTPH1 sensitizes breast cancer cells to Lap by disrupting the EGFR-ER interaction **A.** A stimulation of ER nuclear accumulation by PTPH1 is correlated with an enhancement of total and cytoplasmic EGFR expression. Cell fractionation was performed as previously described [21] with a portion of whole cell lysates (WCL) as an input control. The fold-change was obtained by dividing EGFR bands with the corresponding α-Tubulin and expressed as relative to Vector in WCL. **B.** PTPH1 requires phosphatase activity to disrupt the EGFR/ER interaction. Indicated immune-precipitates were subjected to WB analysis with indicated antibodies. Goat EGFR, rabbit ER, and goat PTPH1 antibodies were used for immune-precipitation (IP). All experiments in **A** and **B** were repeated at least 2 times with the representative shown. **C.** PTPH1 requires its catalytic activity to sensitize breast cancer cells to Lap. T47D cells stably expressed with PTPH1 or its mutants were treated with Lap or solvent and analyzed for colony formation (mean ± SD, *n* = 3).

We previously demonstrated that PTPH1 increases breast cancer sensitivity to anti-estrogens by catalyzing ER/Y537 dephosphorylation [21]. Since PTPH1 decreases EGFR/Y1173 phosphorylation, we next examined if PTPH1 requires its catalytic activity to sensitize breast cancer cells to TKIs. T47D cells stably expressed with PTPH1 (Figures 3A/3B) [21] were assessed for TKI-induced growth inhibition as described above. Interestingly, we found that only expressed PTPH1, but not its phosphatase-deficient mutants, significantly increases the growth-inhibition by two TKIs (Figure 3C; Supplementary Figures 3A/3B). These results indicate that PTPH1 depends on its catalytic activity to sensitize breast cancer cells to TKIs.

Because EGFR-ER interaction is associated with TAM resistance in breast cancer [10] and EGFR/ER signal cross-talk is bidirectional [12], we next tested if PTPH1 enhances the TKI-induced growth-inhibition by disrupting the EGFR-ER complex. WB analyses of anti-EGFR or ant-ER immunoprecipitates revealed their complex-formation as previously reported [10]. This complex, however, is disrupted by PTPH1 (but not by its mutant S459A) overexpression as demonstrated by EGFR IP (Figure 3B), indicating an inhibitory role of the tyrosine dephosphorylation in EGFR interaction with ER. Consistent with our previous findings [21], cell fractionation analysis showed that PTPH1 depends on its phosphatase activity to increase ER nuclear accumulation (Figure 3A). Interestingly, PTPH1 also stimulates EGFR protein expression, especially in cytoplasmic compartment (Figure 3A). PTPH1 proteins are also detectable in EGFR and ER precipitates and an inhibition of the EGFR-ER interaction by PTPH1 expression couples with its relocation from the ER precipitates to the EGFR complexes (Figure 3B). Since the tyrosine kinase EGFR is a natural substrate of tyrosine phosphatases such as PTPH1 [27], one mechanism for the EGFR-ER-complex disruption by PTPH1 may result from its competitive binding and consequently replacing ER for interaction with EGFR. This conclusion is supported by increased EGFR and decreased ER levels in PTPH1 precipitates in PTPH1 overexpressed cells as compared to those transfected with vector, *albeit* the effect on ER less substantial (right, Figure 3B). The EGFR competitive-binding activity of PTPH1 requires its catalytic activity and correlates with its sensitizing effect to TKIs (Figures 3B/3C; Supplementary Figures 3A/3B). These results together indicate that PTPH1 increases the growth-inhibitory activity of TKIs by disrupting the EGFR-ER complex through its EGFR binding activity via a competitive enzyme-substrate interaction.

### A nuclear-localization defective ER has a higher binding activity with EGFR and confers the resistance to TKIs

**Figure 4:**
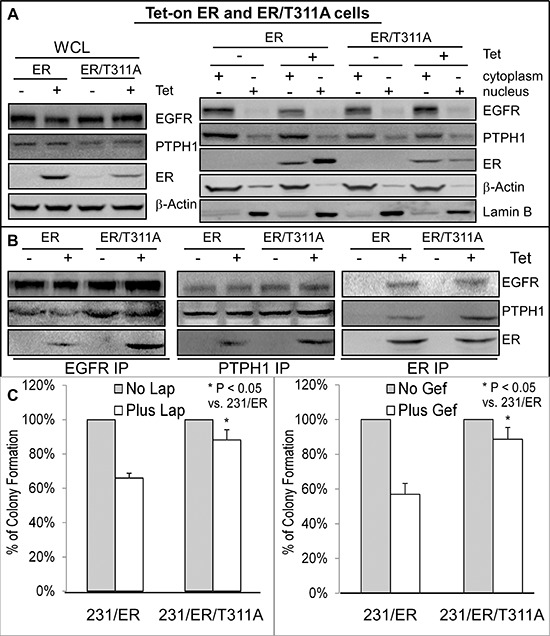
Increased ER-EGFR interaction couples with resistance to Lap-induced growth inhibition **A, B.** EGFR has a higher binding affinity to the cytoplasmic ER/T311A mutant. Tet-on ER and ER/T311A 231 cells were cultured with or without Tet for 24 h and then subjected to cell fractionation and IP analyses (by using the same antibodies as in Figure 3). Every experiment in **A** and **B** was repeated at least 2 times with the representative shown. **C.** ER/T311A expressed 231 cells are more resistant to TKIs than those expressed with ER. Indicated cells in the presence of the same concentration of Tet were cultured with Lap or Gef and effects on colony formation were assessed (mean ± SD, *n* = 3).

One explanation for the PTPH1 capacity to disrupt the EGFR/ER complex may be due to its stimulation of ER nuclear accumulation as a result of the ER/Y537 dephosphorylation [21]. This would lead to decreased levels of extra-nuclear ER proteins available for interacting with cytoplasmic EGFR [10]. To demonstrate if an alteration of cellular ER localization alone is sufficient to regulate its interaction with EGFR, we used the Tet-on system to express ER and its mutant ER/T311A in ER negative 231 cells [28]. Thr311 in the hormone-binding domain of ER is required for ER nuclear localization and its mutation to Ala (ER/T311A) reduces ER nuclear levels [28, 29]. Results (Figure 4A) showed that although the ER/T311A is expressed to a lesser extent than ER after Tet addition in whole cell lysates (WCL), its relative level in the cytoplasm over the nucleus is higher than that of ER. Analysis of anti-EGFR precipitates show a greater amount of the EGFR-ER complex-formation in ER/T311A than ER expressed cells (Figure 4B), indicating that the cytoplasmic ER has a higher binding affinity to EGFR. Consistent with this notion, the cytoplasmic PTPH1 also binds more ER/T311A than ER (PTPH1 IP, Figures 4A/4B). Because ER and ER/T311A are expressed at different levels after Tet addition {Figure 4A, likely as a result of their different stability and/or different localizations [21]}, they were transiently co-transfected with Myc-EGFR in 293T cells and their EGFR binding activities were further analyzed. WB analysis of the Myc precipitates showed that Myc-EGFR binds increased levels of the cytoplasmic ER/T311A but decreased amounts of the nuclear GFP-ER/Y537F [30] as compared to their respective wild-type (WT) proteins (Supplementary Figure 3C). These results further demonstrate that EGFR has a higher binding affinity to the cytoplasmic ER and a decreased activity in interacting with the nuclear ER. Importantly, ER/T311A expressed cells are more resistant to both TKIs than cells expressed with ER (Figure 4C). These results further demonstrate that the ER-bound EGFR is less effective than its free form as a therapeutic target for TKIs in breast cancer.

### The therapeutic target activity of EGFR depends on its interaction with PTPH1 and on its Y1173 phosphorylation

**Figure 5:**
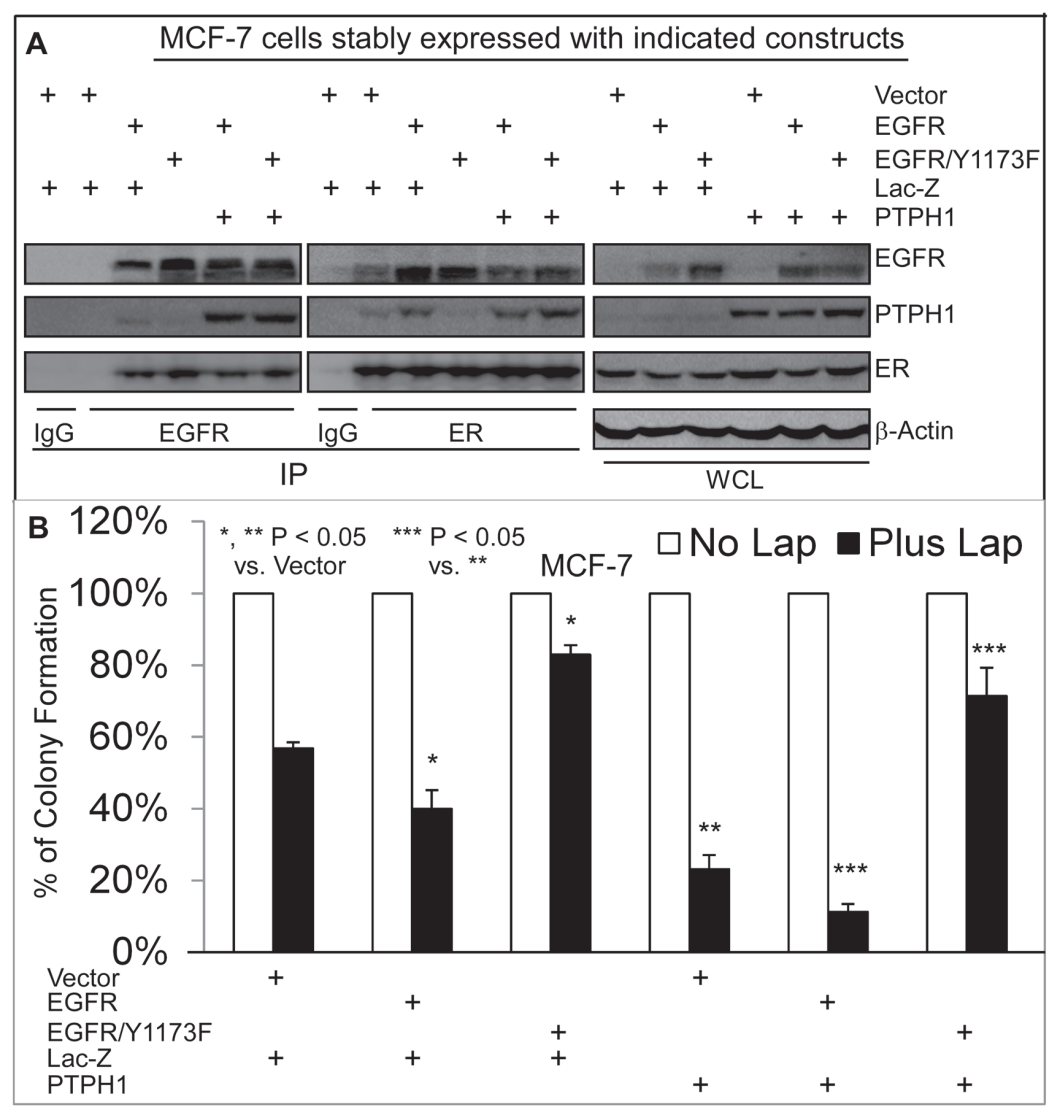
Y1173 is required for breast cancer sensitivity to Lap, for EGFR interaction with endogenous PTPH1, and for PTPH1-induced sensitization **A.** Y1173 is required for EGFR to bind endogenous PTPH1 but not ER. MCF-7 cells were stably co-expressed with indicated constructs by transfection and retroviral infection, which were analyzed by IP/WB with a portion of WCL as an input control (antibodies used as described in Figure 3). Every experiment was repeated at least 2 times with the representative shown. **B.** Y1173 is required for Lap-induced growth inhibition and for PTPH1-induced sensitization. Indicated cells were cultured with Lap (5 μM) or solvent and effects on colony formation were assessed (mean ± SD, *n* = 3).

Small molecule TKIs exhibit therapeutic activities by suppressing EGFR phosphorylation resulting in decreased cell growth and proliferation [4]. Our results however showed that PTPH1 decreases EGFR/Y1173 phosphorylation and increases breast cancer sensitivity to TKI-induced growth inhibition. We therefore determined if Y1173 is required for the growth-inhibitory activity of TKIs through regulating EGFR interaction with PTPH1 and/or ER. MCF-7 cells were stably expressed with EGFR and its Y1173F mutant. Thereafter, cells were further expressed with and without PTPH1 by retroviral infection through a separate antibiotic selection to determine if PTPH1 expression requires Y1173 to confer the sensitization. Results showed that the forced-EGFR expression increases the growth-inhibition by Lap, whereas the EGFR/Y1173F transfection confers the resistance, as compared with the vector transfection (Figures 5A/5B). Similar results were obtained in T47D cells and/or after the treatment with Gef (Supplementary Figures 3D–3F), indicating that Y1173 is required for breast cancer sensitivity to TKIs. Consistent with the diminished EGFR binding activity of the phosphatase-deficient PTPH1/S459A (Figure 3B), analysis of EGFR precipitates show that the ectopically expressed EGFR/Y1173F failed to interact with endogenous PTPH1 as compared to the WT EGFR (Figure 5A). However, ER is able to bind EGFR and EGFR/Y1173F in ER precipitates, both of which are suppressed by the ectopically expressed PTPH1 (Figure 5A). In EGFR/Y1173F expressed cells, ER also fails to interact with endogenous PTPH1, suggesting that ER may bind endogenous PTPH1 through EGFR. Most importantly, PTPH1 overexpression further sensitizes cells expressed with EGFR but confers the resistance in those expressed with the Y1173F mutant (Figure 5B; Supplementary Figure 3F). PTPH1 silencing also increases levels of p-EGFR/Y1173 and attenuates the growth-inhibition by TKIs in cells expressed with EGFR but not with its Y1173 mutant (Supplementary Figures 4A–4C). Together, these results indicate that the therapeutic target activity of EGFR depends both on Y1173 phosphorylation and on its interaction with PTPH1.

### PTPH1 increases EGFR protein stability by catalyzing EGFR/Y1173 de-phosphorylation

Tyrosine phosphorylation triggers EGFR degradation through an internalization process [2, 31]. Analysis of whole cell lysates showed that PTPH1 expression increases endogenous (Figure 3A) and transfected EGFR protein expression (Figure 5A). We next examined if PTPH1 increased EGFR protein stability by decreasing its phosphorylation at Y1173. T47D cells stably expressed with PTPH1 and its phosphatase-inactive PTPH1/S459A mutant were cultured with cycloheximide (CHX), a protein synthesis inhibitor. Endogenous EGFR protein expression was examined by direct WB analysis. Results (Supplementary Figure 5A) show that PTPH1 significantly increases the EGFR stability as compared to its S459A mutant, indicating that PTPH1 depends on its catalytic activity to stabilize EGFR protein. Analysis of MCF-7 cells further demonstrated that PTPH1-forced expression stabilizes the ectopically expressed EGFR but not EGFR/Y1173, and the EGFR/Y1173F is more stable than its WT counterpart (Supplementary Figure 5B). Transient co-transfection in 293T cells further demonstrated that PTPH1 inhibits EGFR (but not its Y1173F mutant) ubiquitination and proteasome-dependent degradation, and PTPH1/S459A is less effective in these actions (Supplementary Figure 5C). In addition, elevated PTPH1 in breast cancer tissues correlates with increased EGFR protein expression (Supplementary Figures 6A/6B). These results together demonstrate that PTPH1 increases EGFR protein stability/expression by decreasing Y1173 phosphorylation thereby inhibiting its proteasome-dependent degradation.

### PTPH1 increases the membranous EGFR and nuclear ER levels, and confers breast cancer sensitivity to a combined therapy of TKIs with an anti-estrogen

PTPH1 belongs to the non-receptor PTP family [27] and is mostly localized to the cytosol [20, 21] (Figures 3/4). EGFR is a transmembrane receptor and its translocation to the nucleus is associated with resistance to EGFR targeted therapies [2, 32], whereas its membranous accumulation appears to be necessary for the efficacy of anti-EGFR therapy [33]. In contrast, the nuclear receptor ER exerts its biological functions both through its nuclear and extra-nuclear activities [6]. Our results showed that PTPH1 increases breast cancer sensitivities to TKIs through disrupting the ER-EGFR interaction by catalyzing EGFR/Y1173 de-phosphorylation (Figures 1–5). Moreover, PTPH1 stimulates ER nuclear accumulation and increases breast cancer sensitivity to anti-estrogens [21]. These results together indicate that a physical interaction between EGFR and ER in low PTPH1 expressed cells may restrain or limit their therapeutic target activities through an alteration of their cellular localization. Elevation of cellular PTPH1 concentrations may restore the natural EGFR/ER cellular distributions by attenuating their complex-formation and consequently confer the sensitivity to combined therapies of TKIs with an anti-estrogen.

**Figure 6:**
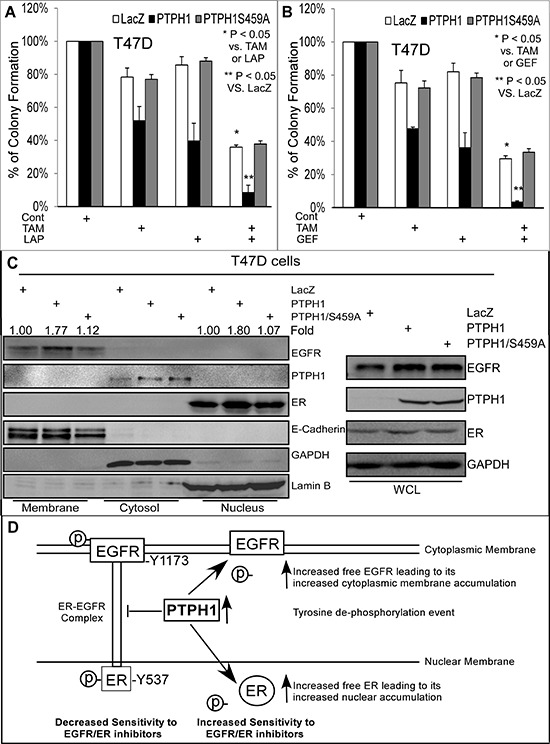
PTPH1 expression sensitizes breast cancer cells to a combined therapy of TKIs with anti-estrogen in association with increased cytoplasmic membranous EGFR and nuclear ER accumulation **A, B.** T47D cells stably expressed with and without PTPH1 or PTPH1/S459A were treated with Gef (1 μM) or Lap (2.5 μM) in combination with TAM (50 nM) for about 2 weeks and resultant colonies were counted (mean ± SD, *n* = 3). **C.** PTPH1 depends on its catalytic activity to increases cytoplasmic membranous EGFR and nuclear ER accumulation. T47D cells with and without stably expressed PTPH1 or PTPH1/S459A were subjected to cell fractionation analyses as described [34]. The fold change was obtained by dividing EGFR (left) or ER (right) bands with the corresponding E-cadherin (EGFR) or Lamin B (ER) and expressed as relative to LacZ. Similar results were obtained from at least two separate experiments with the representative shown. **D.** Our experimental model indicates that PTPH1 may sensitize breast cancer cells to EGFR/ER inhibitors by decreasing tyrosine de-phosphorylation of EGFR and ER leading to a disruption of their complex and a restoration of their physiological localizations. According to this model, the EGFR-ER complex confers an intrinsic resistance to EGFR and ER inhibitors, whereas their non-bound forms are better targets for therapeutic intervention.

To test this possibility, T47D cells stably expressed PTPH1 (or its mutant) were analyzed for their sensitivity to combined therapies of TKIs with the ER inhibitor tamoxifen (TAM). To further dissect the distribution of EGFR and ER in different cellular compartments, a recently published protocol [34] was used to prepare proteins from the membrane, cytosol, and nucleus. Results (Figures 6A/6B) showed an increased growth-inhibition by the combined treatment of Lap or Gef with TAM in Vector-transfected cells over either inhibitor alone. A forced PTPH1, but not PTPH1/S459A, expression significantly increases the growth-inhibition by the combination compared to either alone (Figures 6A/6B). Cell fractionation analysis showed that the PTPH1 expression stimulates both the membranous EGFR and the nuclear ER accumulation, while its catalytic deficient mutant lacks such activities (Figure 6C). Similar results were also obtained in MCF-7 cells (Supplementary Figures 6C/7A/7B). The sensitization effect of PTPH1 to the combined therapy was further demonstrated by cell viability assays (Supplementary Figures 7C–7F). Experiments with stably co-transfected MCF-7 cells further showed that PTPH1 increases the membranous EGFR and the nuclear ER as demonstrated by cell fractionation and immune-staining analyses (Supplementary Figures 8A–8C). Furthermore, a more substantial elevation of PTPH1 protein-expression in breast cancer tissues appears to correlate with a significant increase in the membranous EGFR (Supplementary Figure 6A, left) [33]. These results together indicate that PTPH1 may confer breast cancer sensitivity to combined therapies of TKIs with TAM by decreasing tyrosine phosphorylation of both EGFR and ER. This will lead to a disruption of their interaction and consequently result in a restoration of their physiological cellular localization (Figure 6D).

## DISCUSSION

Protein-protein interaction is increasingly realized to play a critical role in life-important events [35] and is now considered as a cancer target for therapeutic intervention [18, 36]. However, a complex-formation of therapeutic targets can also be a resistant factor for targeted therapies [10, 37] and mechanisms involved are mostly not understood. Our results, together with the published reports, suggest that a tyrosine-dephosphorylation event is a switch to turn off the EGFR-ER inhibitory complex. This will increase the membranous EGFR concentration and the nuclear ER accumulation leading to optimization of their therapeutic target activities (Figure 6D). This model is based the fact that increased ER-EGFR interaction, enhanced extra-nuclear ER accumulation [10, 11], and elevated EGFR protein expression [38] are all associated with breast cancer resistance to anti-estrogens. Moreover, ER/Y537 phosphorylation is required for ER nuclear export [30] and PTPH1 dephosphorylation of ER/Y537 stimulates ER nuclear accumulation and increases breast cancer sensitivity to anti-estrogens *in vitro* and *in vivo* [21]. Furthermore, tyrosine dephosphorylated EGFR is more stable and resistant to proteasome-dependent degradation [31, 39], whereas increased nuclear EGFR activity is associated with resistance to EGFR-targeted therapies [2, 32]. Because PTPH1 dephosphorylates EGFR and ER [17, 21] (Figure 1), tyrosine de-phosphorylation may be the primary enzymatic event that disrupts the EGFR-ER complex. This will result in their free forms back to the original cellular compartments where their therapeutic target activities are maximized (Figure 6D). This conclusion is further supported by the fact that the nuclear-localization-defective ER/T311A mutant has a higher EGFR binding affinity and confers the resistance to TKIs independent of PTPH1 (Figure 4). Therefore, a complex formation of EGFR with ER may be an intrinsic resistant factor both to TKIs and anti-estrogens. This inhibitory complex can be switched off by PTPH1-induced tyrosine de-phosphorylation thereby promoting their free forms back to their physiological cellular compartments leading to an increase in breast cancer sensitivity to their targeted therapies (Figure 6D).

Our results with PTPH1 analyses reveal a novel mechanism for combined applications of TKIs with anti-estrogens in breast cancer targeted therapy. TKI decreases p-EGFR/Y1173 levels (Figure 1) and EGFR/Y1173F is more stable and exists to a lesser extent than its WT counterpart in ER precipitates (Figure 5A; Supplementary Figure 5B). These effects mimic actions of PTPH1 expression and activation (Figures 1/3B/5A). Furthermore, anti-estrogens increase ER nuclear accumulation [40], whereas in TAM resistant breast cancer there is increased EGFR-ER binding and extra-nucleus ER activity [10]. In addition, the ER/Y537F mutant that is exclusively localized in the nucleus has a decreased binding affinity to EGFR (Supplementary Figure 3C) [30]. These effects also resemble those occurring in cells overexpressed with PTPH1 protein (Figures 3A/3B/5A). Therefore, the sensitizing effect of PTPH1 on the growth inhibition by TKIs (Figure 2) and by anti-estrogens [21] would indicate their combination advantage. Our findings may explain why a combination of TKIs with anti-estrogens is a better approach to suppress breast cancer growth as demonstrated in preclinical [11, 41] and clinical studies [42]. Our results in ER and ER/T311A expressed cells further indicate that strategies that attenuate the EGFR-ER interaction and/or restore their physiological cellular distributions independent of PTPH1 may also be able to increase the therapeutic response to TKIs and/or anti-estrogens. In addition, PTPH1 may increase breast cancer therapeutic response to Her-2 inhibitors. This is because the Her-2-ER interaction is also associated with the anti-hormone resistance [43, 44] and PTPH1 can decrease Her-2/Y877 phosphorylation (data not shown) and increase breast cancer sensitivity to the EGFR/Her-2 dual inhibitor Lap (Figure 2). Since PTPH1 is overexpressed in about 50% primary breast cancer [20], further investigation are warranted to determine if patients with PTPH1-overexpressed breast cancer will respond better to combination therapies of TKIs with anti-estrogens.

Several PTPs dephosphorylate EGFR at Y1173 (alone or together with other residues), including SHP-1 (*PTPN6*) [45], receptor-type RPTP-κ [46], and *PTPN2* [47]. Both RPTP-κ and *PTPN2* knockdown increase levels of p-EGFR/Y1173 and of p-ERKs [46, 47], indicating a tumor suppressor activity of these PTPs and/or a mitogenic function of EGFR/Y1173 phosphorylation. However, studies also showed that EGFR/Y1173 phosphorylation can confer a growth-inhibitory signal by a methylation-associated cross-talk with EGFR/Arg1175 [22]. Moreover, elevated levels of p-EGFR/Y1173 in primary lung cancer are associated with a poorer clinical response to anti-EGFR therapies [23]. These results are consistent with the oncogenic activity of PTPH1 [16, 17, 19–21] and with its sensitization effect to TKIs via dephosphorylating EGFR/Y1173 (Figures 1–3). Furthermore, PTPH1 dephosphorylates EGFR/Y1173 *in vitro* [17] and negatively regulates p-EGFR/Y1173 levels by both overexpression and silencing (Figure 1). This suggests that oncogenic PTPH1 in breast cancer is a physiologically important mechanism to counteract the growth-inhibitory signal of p-EGFR/Y1173 and the intrinsic resistance to TKIs (Figure 2). PTPH1 and EGFR are co-overexpressed in breast cancer tissues and their expression-levels are both significantly higher in Her-2 positive breast tumors (Supplementary Figure 6 and data not shown). These results would indicate a specific combination advantage of TKIs with anti-estrogens in Her-2 positive breast cancer. Additional studies are needed to investigate if PTPH1 increases the growth-inhibitory activity of TKIs *in vivo* and whether elevated PTPH1 in clinical breast cancer correlates with decreased p-EGFR/Y1173 levels.

## MATERIALS AND METHODS

### Plasmids, constructs, and cell lines

MCF-7, T47D, MDA-MB-231 (231) and 293T cells were obtained from ATCC between 2011 and 2014, and are maintained as described [21] but no authentication was done by the authors. HA-tagged PTPH1 and its phosphatase-deficient mutant PTPH1/DA were kindly provided Dr. N. K. Tonks [13, 24] and used previously in our laboratory [16, 20]. HA-tagged PTPH1/S459A was generated as previously described in our laboratory [17]. The pLenti6/Block-iT system was used to clone sequences for shRNAs against luciferase and PTPH1 (shLuc and shPTPH1) as described [16, 17, 20]. Human EGFR cDNA and its Y1173Y mutant were provided by Mien-Chie Hung [22] and were sub-cloned into pLHCX retroviral vector as previously described [48]. Plasmids GFP-ERα and its Y537F mutant were described previously [30] and were used in our recent publication [21].

### Antibodies and other reagents

Antibody against PTPH1 (mouse) was kindly provided by Dr. N. K. Tonks. Other antibodies used in this study were purchased from Santa Cruz Biotechnology (Santa Cruz, CA). These include Anti-GAPDH (sc-47724), anti-ERα (rabbit, sc-543), anti-ERα (mouse, sc-8002), anti-EGFR (rabbit, sc-03) anti-EGFR (goat, sc-03G), anti-p-EGFR/Y1173 (goat, sc-12351), anti-PTPH1 (goat, sc9789), anti-α-Actinin (sc-17829), anti-β-actin (sc-47778), anti-ubiquitin (sc-8017), anti-Lamin B (sc-6217), anti-phospho-Tyr (sc-18182 and sc-508), anti-c-Jun (sc-44), anti-GFP (sc-9996), anti-α-Actinin (sc-17829), and anti-α-Tubulin (sc-6199). The dual EGFR/Her2 inhibitor lapatinib (Lap) and the EGFR inhibitor gefitinib (Gef) were obtained Selleckem. Cycloheximide (CHX), estrogen (E2), EGF, and 4-hydroxy-TAM (TAM) were purchased from Sigma. Anti-p-EGFR/Y1173 (rabbit, 4407L) was obtained from Cell Signaling and anti-E-cadherin (610181) was from BD Biosciences. Cell culture medium and serum were provided by Gibco.

### Gene expression and silencing

PTPH1, PTPH1/DA, and PTPH1/S459A stably expressed T47D cells were generated by retroviral infection [20, 21]. Tetracycline inducible (Tet-on) system (Invitrogen) was used to express PTPH1 in MCF-7 and 231 cells [20, 49, 50]. Tet-ER and Tet-ER/T311A 231 cells were generated previously [28]. To induce gene expression by the Tet-on system, cells were typically incubated with and without Tet for 24 h and then used for various experiments. Silencing of PTPH1 in MCF-7 and T47D cells was achieved by infection with lenti-viruses containing shPTPH1 or control shLuc, followed by antibiotic selection as described previously [20, 21]. EGFR and its Y1173F mutant was stably expressed by G418 selection and resistant cells were further infected with retroviruses (pLHCX vector or pLHCX-PTPH1) to co-express PTPH1 [48].

### Cell fractionation, immunoprecipitation, immunoblot analysis, and immune-staining

For cytosol/nuclear cell fractionation analysis, the previous published protocol from this lab was used [20]. For the membrane/cytosol/nuclear cell fractionation, we followed the protocol published by Rockstroh et al [34] (the method 2). The same protein amount from each group was used for immunoprecipitation (IP) analysis. An aliquot of whole cell lysates (WCL) was used as an input control. Briefly, cells were washed with cold PBS and lysed in modified RIPA buffer (50 mM Tris-HCL, pH 7.5, 1 mM phenylmethylsulfonyl fluoride, 1 mM dithiothreitol, 10 mM sodium fluoride, 1 μg/ml aprotinin, 1 μg/ml leupeptin, 1 μg/ml pepstatin containing 1% Nonidet P-40 as described previously [20]. Cleared lysates were then incubated with indicated antibodies or IgG overnight at 4°C. Precipitates were then washed and pellets were re-suspended in 2 × loading [28]. For direct WB, cells were directly lysed in 1 × loading buffer. After heating, samples were separated on SDS-PAGE and the rest of the procedure was the same as previously described [28]. For immune-staining analysis, cells were plated on coverslips and fixed in 3.7% formaldehyde. Cells were then permeabilized in a buffer containing 0.5% Triton X-100 and 0.5% NP40, and then incubated with a blocking buffer (PBS containing 3% bovine serum) [28].

### Colony formation, viability assay, treatments with EGFR and ER inhibitors, and ER stability studies

For colony formation, breast cancer cells stably engineered to overexpress or silence PTPH1 (and/or EGFR) were plated (500–1000 cells/ml) in 6 well plates. On the next day, cells were treated with indicated inhibitors. Approximately 2 weeks later, colony formations were stained, photographed, and manually counted following the previous protocol [20]. To assess cell viability, cells were continuously treated with inhibitors for 72 h and viable cells (not stained with trypan blue) were counted with a hemocytometer as previously described [21]. To assess EGFR protein stability, T47D or MCF7 cells stably expressed with PTPH1 and/or EGFR (their mutants) were treated with CHX (100 μg/ml) for various time and lysates were collected for WB analysis.

### Statistical analysis

Results were analyzed by Student's *t*-test, unless specified.

## SUPPLEMENTARY FIGURES


