# Supplementary material for: Tyrosine dephosphorylation enhances the therapeutic target activity of epidermal growth factor receptor (EGFR) by disrupting its interaction with estrogen receptor (ER)
Source: Oncotarget. 2015 Apr 10;6(15):13320–33. doi: 10.18632/oncotarget.3645 (PMC4537017; doi:10.18632/oncotarget.3645)
Supplement: Supplementary file 1 [file oncotarget-06-13320-s001.pdf]

SUPPLEMENTARY FIGURES

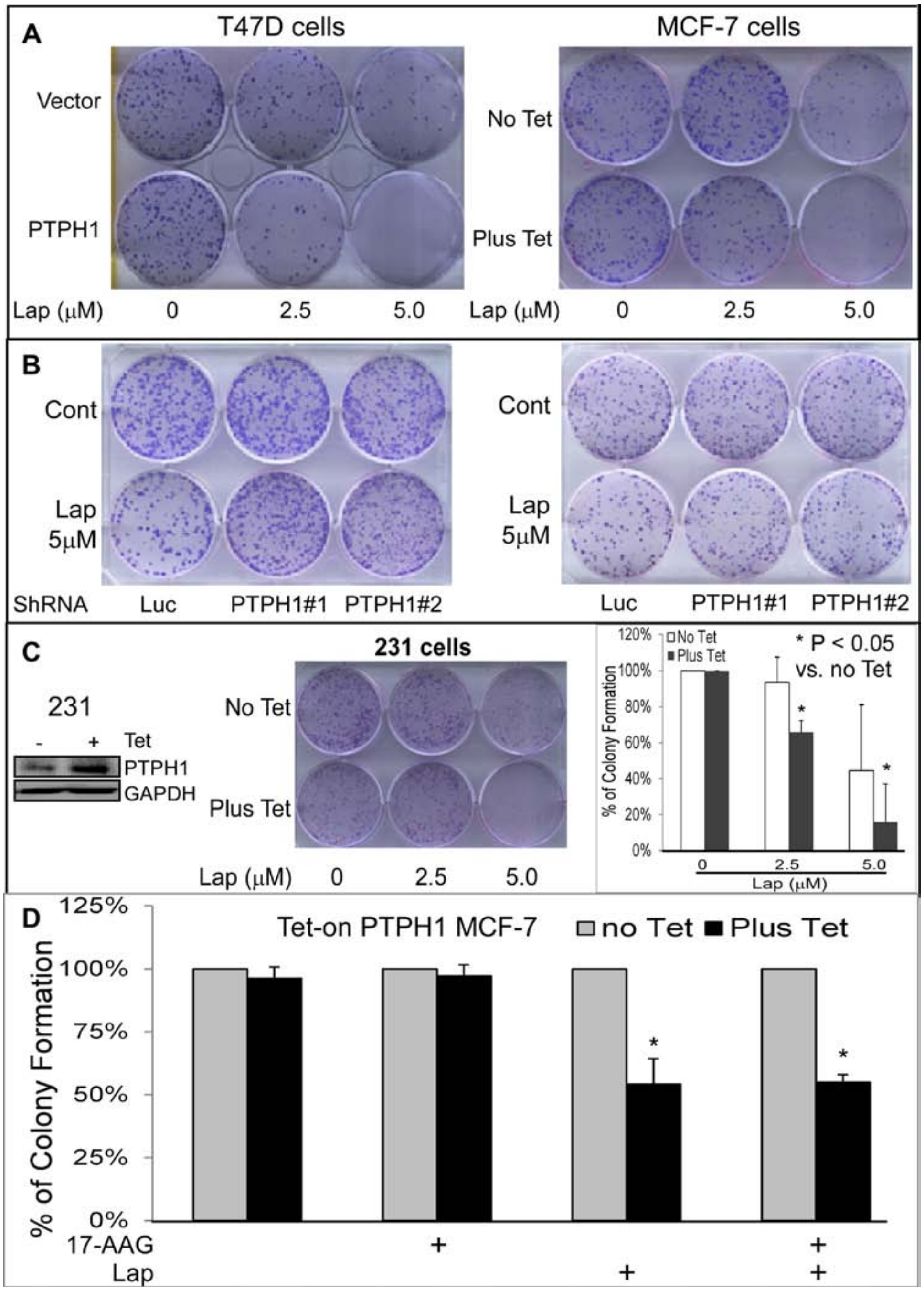

Supplementary Figure S1: PTPH1 confers breast cancer cell sensitivity to Lap but not to 17-AAG. A. PTPH1 overexpression increases the growth inhibition by Lap. Cells were cultured continuously with Lap or solvent (with or without Tet for Tet-on PTPH1 MCF-7 cells) for approximately 2 weeks and plates were then fixed and photographed. **B.** PTPH1 depletion confers resistance to Lab-induced growth inhibition. PTPH1 stably depleted (or control shLuc) cells were incubated with Lap or solvent for approximately 2 weeks and plates were then fixed and photographed. **C.** PTPH1 overexpression increases the Lap-induced growth inhibition in 231 breast cancer cells. Tet-on PTPH1 231 cells were continuously treated with Lap or solvent, and assessed for colony formation. PTPH1 expression was shown in the left panel, typical colony formation was shown in the middle, and summarized results of colony formation were presented in the right panel (mean ± SD, *n* = 3). **D.** PTPH1 overexpression does not increase the 17-AAG induced growth inhibition. Tet-on MCF-7 cells were cultured in the absence and presence of Tet with 2.5 M of Lap with and without 0.1 M of 17-AAG for about 2 weeks and resultant effects on colony formation were determined (mean ± SD, *n* = 3)

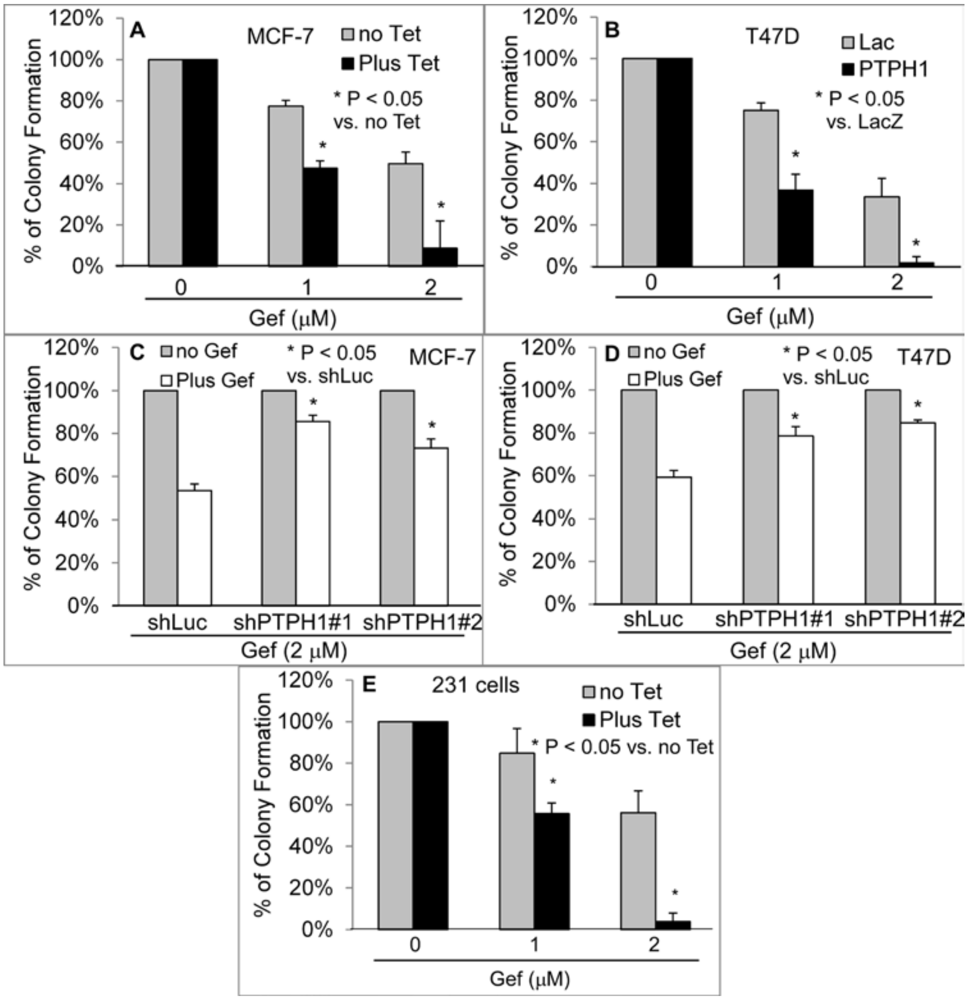

Supplementary Figure S2: PTPH1 expression increases and its depletion decreases breast cancer cell sensitivity to Gef-induced growth inhibition. A, B. PTPH1 expression confers sensitivity of breast cancer cells to Gef-induced growth inhibition. Indicated cells were cultured with Gef or solvent for approximately 2 weeks and colony formation assay was performed. Results are means of three experiments (± SD). **C, D.** PTPH1 knockdown increases breast cancer cell resistance to Gef-induced growth inhibition. PTPH1 stably depleted cells were cultured with Gef or solvent and effects on colony formation were performed as described above (mean ± SD, $n = 3$). **E.** PTPH1 expression increases sensitivity of 231 cells to Gef-induced growth inhibition. Cells were cultured with Gef in the absence or presence of Tet and effects on colony formation were assayed as described above (mean ± SD, $n = 3$).

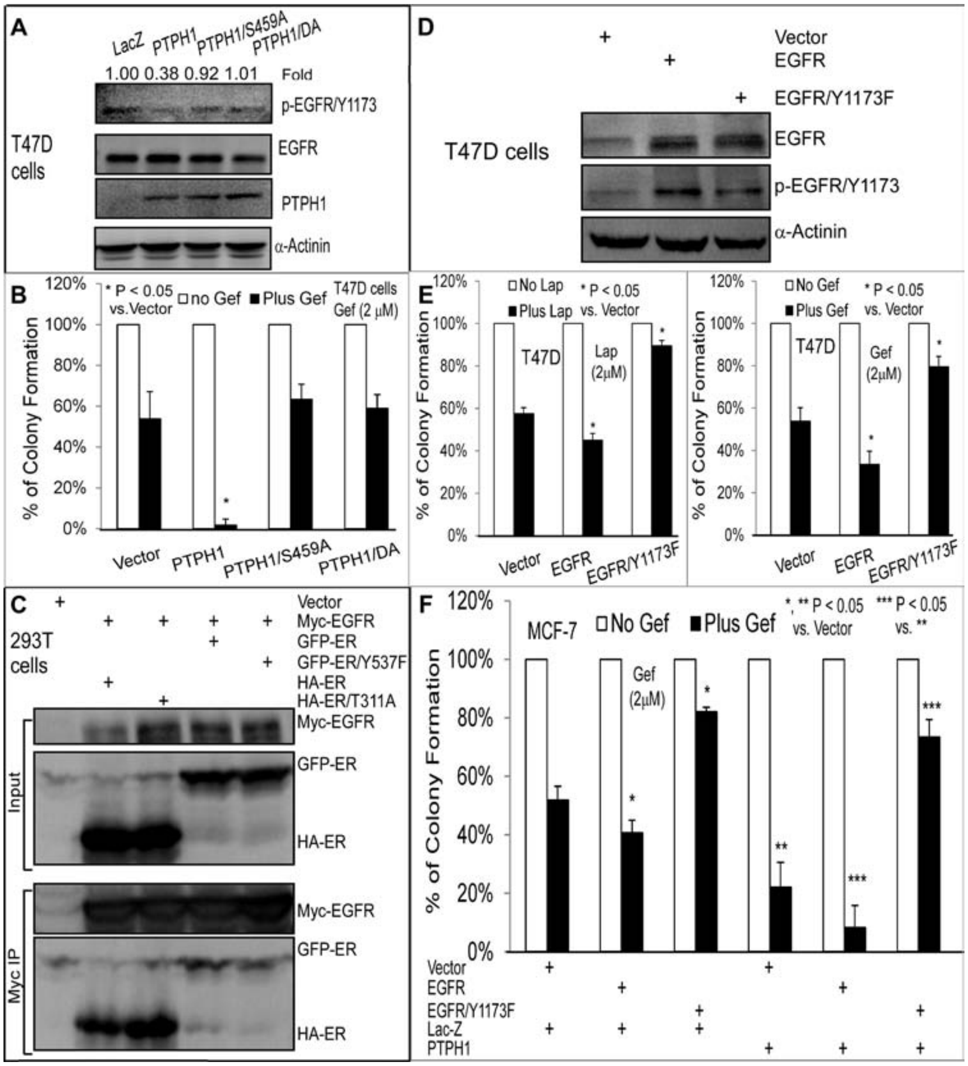

Supplementary Figure S3: The required role of PTPH1 catalytic activity and EGFR/Y1173 in breast cancer sensitivity to Gef/Lap-induced growth inhibition. **A, B.** PTPH1 (and its mutant) stably expressed T47D cells were analyzed for protein expression/phosphorylation by WB (**A**) and for Gef-induced growth inhibition by colony formation (**B**). The fold **A** was obtained by dividing p-EGFR/y1173 bands with the corresponding EGFR and expressed as relative to LacZ. Similar results were obtained from at least two separate experiments. Results (**B**) are means of three experiments (± SD). **C.** EGFR binds more cytoplasmic ER/T311A but less nuclear ER/Y537F as compared to their respective wild-type counterparts by transient transfection in 293T cells. Cells were transfected with indicated constructs and analyzed by IP with a mouse Myc antibody. Myc precipitates were analyzed by WB with indicated antibodies (rabbit anti-ER and goat anti-EGFR). Similar results were obtained from at least two separate experiments. **D-F.** EGFR expression increases, but its Y1173F mutant expression (**D**) decreases, breast cancer sensitivity to TKI-induced growth inhibition (**E**) or to the PTPH1-induced sensitization F (mean ± SD, $n = 3$). The residual p-EGFR/Y1173 signal in EGFR/Y1173F stably expressed T47D cells (**D**) may be due to endogenous EGFR phosphorylation (similar results were obtained from at least two separate experiments).

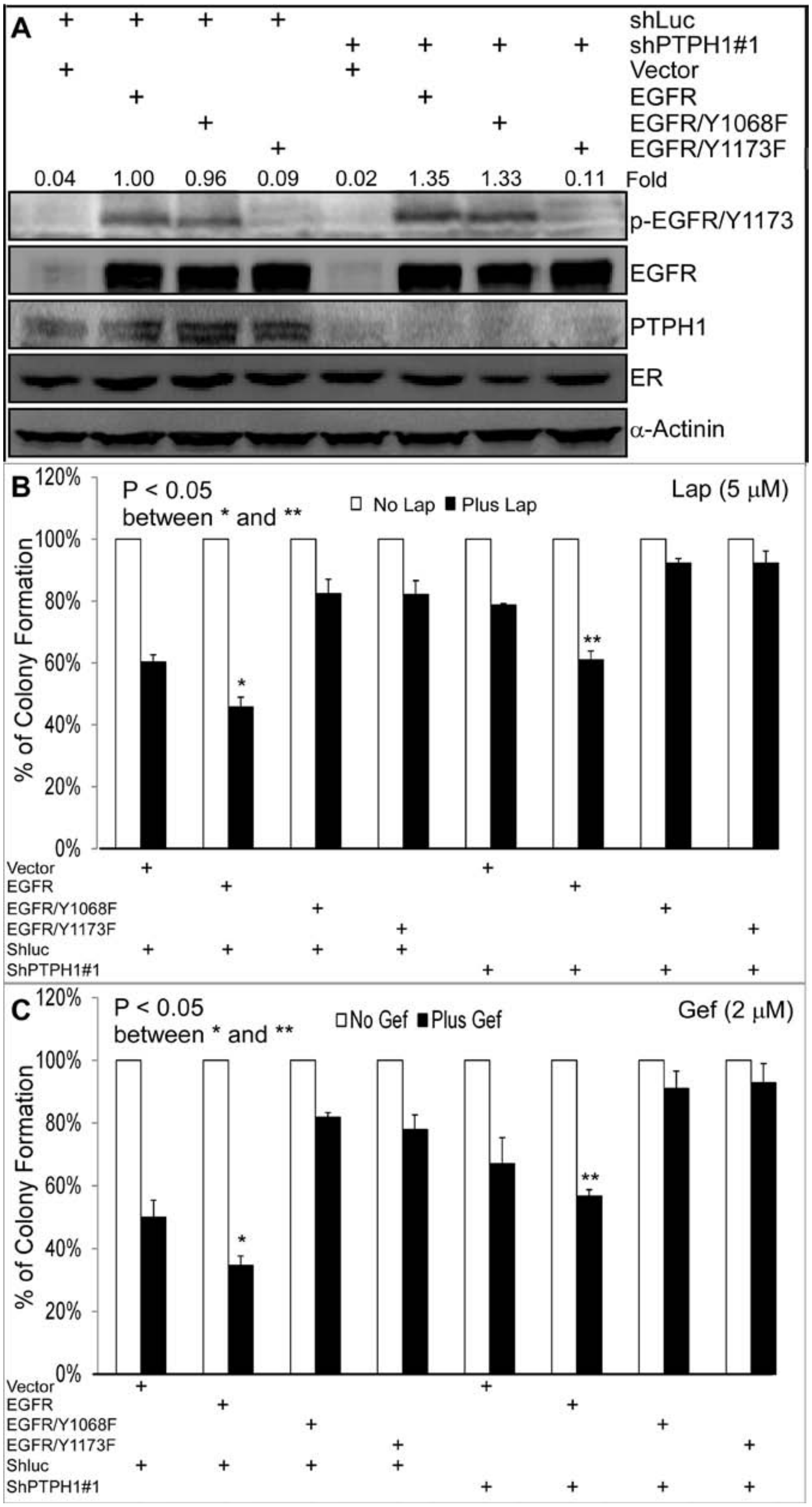

Supplementary Figure S4: PTPH1 knockdown increases levels of ectopically expressed EGFR phosphorylation at Y1173 and attenuated EGFR-expression induced sensitization to TKIs. A. EGFR and its mutant-expressed MCF-7 cells were depleted of endogenous PTPH1 protein by lentiviral mediated shRNA delivery and assessed for protein expression and phosphorylation by WB. The fold change was obtained by dividing p-EGFR/Y1173 bands with the corresponding EGFR and expressed as relative to EGFR/shLuc. Similar results were obtained from at least two separate experiments. **B, C.** indicated engineered MCF-7 cells were cultured with Lap or Gef (or solvent control) for approximately 2 weeks and assessed for colony formation. Results are means of 3 experiments (± SD).

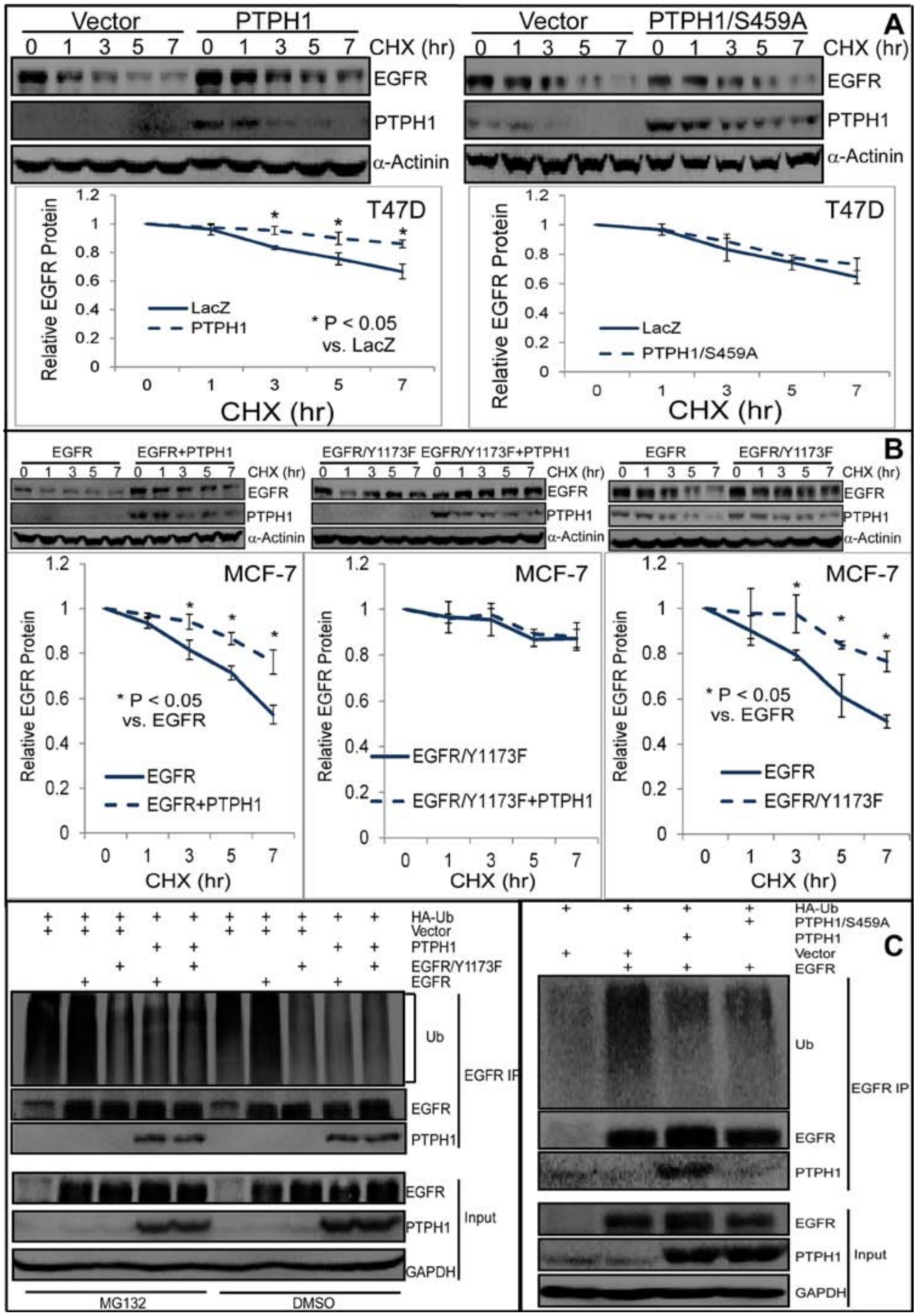

Supplementary Figure S5: PTPH1 increases EGFR protein stability by catalyzing EGFR/Y1173 dephosphorylation. **A.** PTPH1 depends on its catalytic activity to stabilize EGFR protein. T47D cells stably expressed with PTPH1 or PTPH1/S459A were cultured with CHX (100 µg/ml) for indicated time and assayed for protein expression by WB. A representative blot was shown on top and an average of quantitative results by a phospho-Imager from 3 experiments was presented at bottom (± SD). **B.** MCF-7 cells stably co-expressed with EGFR or EGFR/Y1173F together with PTPH1 (or vector) were cultured with CHX for indicated time and analyzed for EGFR protein stability as described above (bottom, mean ± SD, *n* = 3). **C.** 293T cells were transiently expressed with indicated constructs and assessed for protein ubiquitination and expression by IP/WB after treatment with or without MG132 (100 µM) for the last 6 h. A goat anti-EGFR antibody was used for IP and EGFR precipitates were analyzed by WB using indicated antibodies. Similar results were obtained in a separate experiment. Results showed that PTPH1 expression decreases the co-expressed EGFR ubiquitination and degradation that is blocked by MG132 (left). Moreover, the expressed EGFR/Y1173F exhibited decreased ubiquitination independent of the PTPH1 co-transfection. PTPH1/S459A has attenuated effects on EGFR ubiquitination and protein expression (right).

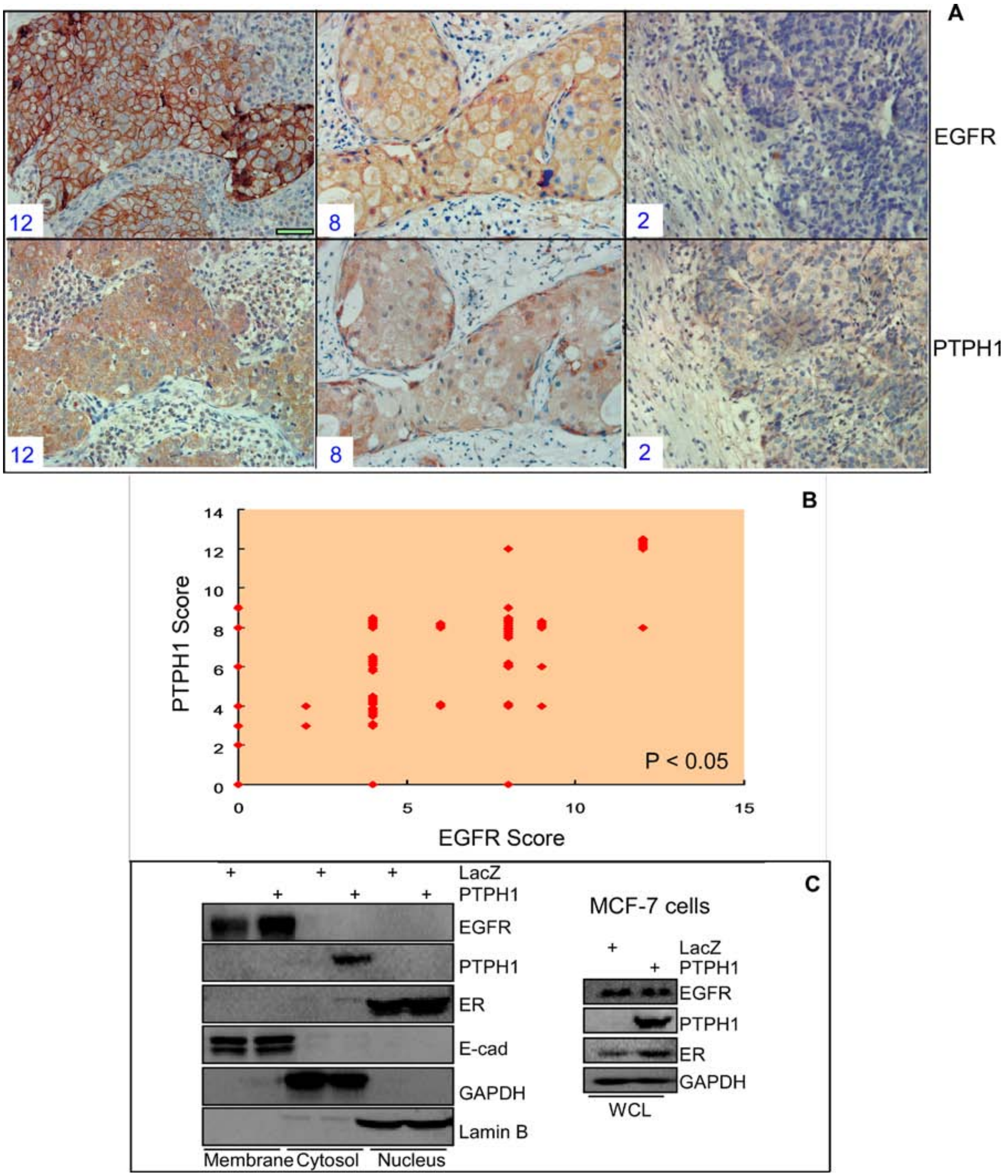

Supplementary Figure S6: Levels of PTPH1 protein expression correlate with EGFR in primary breast cancer tissues and PTPH1 transfection in MCF-7 cells increases the membranous EGFR and nuclear ER accumulation. A, B. a group of primary breast cancer specimens (120 cases) were assessed for PTPH1 and EGFR protein expression by immunohistochemistry (IHC) with the representative images shown in **A** in which the staining score was given. A Person's correlation was reached between increased PTPH1 and EGFR expression (**B**, $P < 0.05$). Please note that higher PTPH1 expression in breast cancer tissues correlates with increased membranous EGFR signal (**A**, left). **C.** engineered MCF-7 cells were analyzed for protein expression and distribution by cell fractionation and WB. Similar results were obtained in a separate experiment.

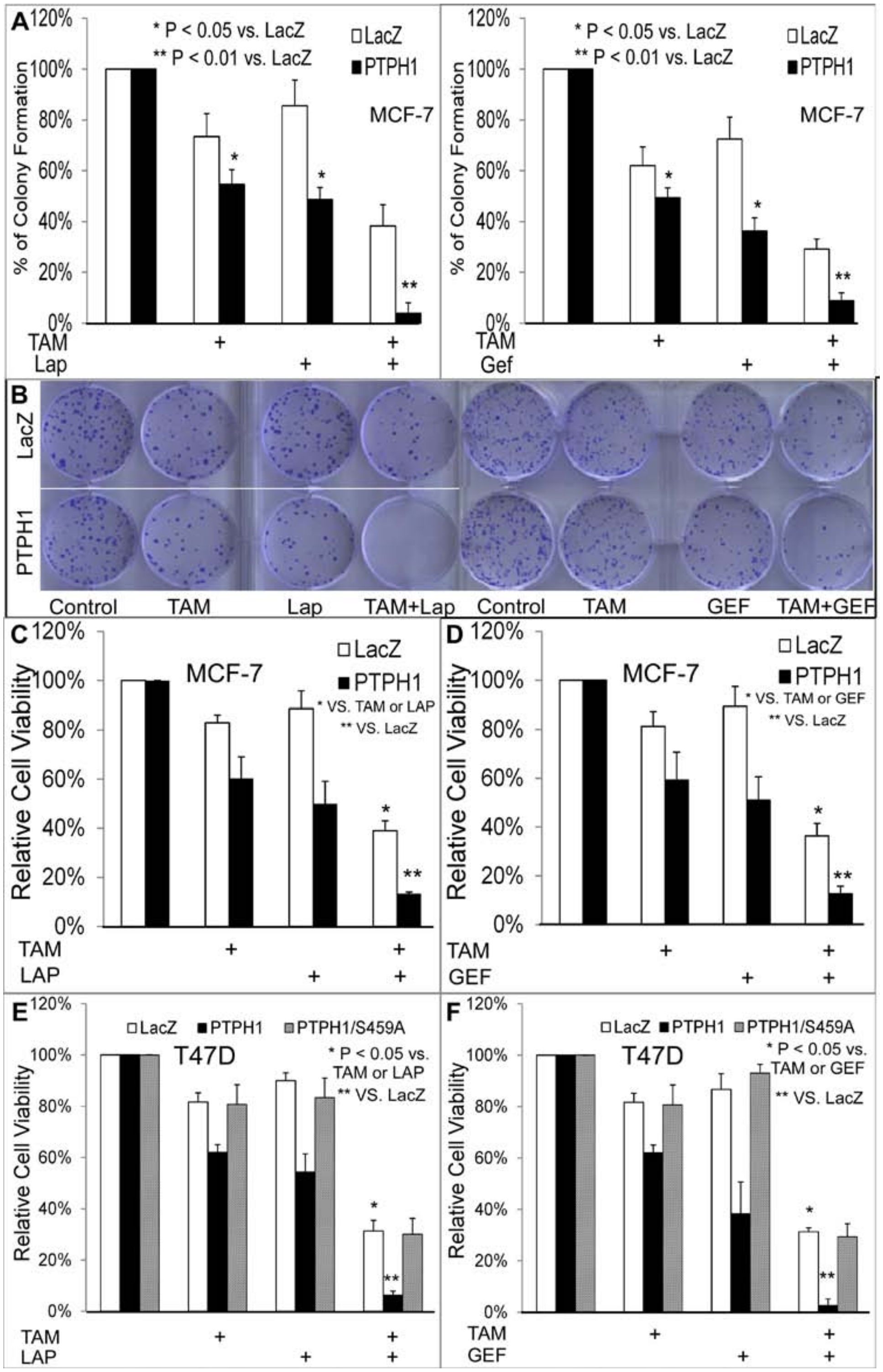

Supplementary Figure S7: PTPH1 expression confers the breast cancer sensitivity to a combination therapy of TKIs with TAM. A, B. MCF-7 cells stably expressed with PTPH1 or vector were treated with Gef (1 μM) or Lap (2.5 μM) in combination with TAM (50 nM) for about 2 weeks and resultant colonies were counted (A, mean ± SD, *n* = 3) or photographed (**B**). **C–F.** indicated cells were incubated with Lap (5 μM) or Gef (2 μM) together with and without TAM (1 μM) for 48 h and cell viability was determined by trypan blue exclusion assays. Results are mean of 3 separate experiments (± SD).

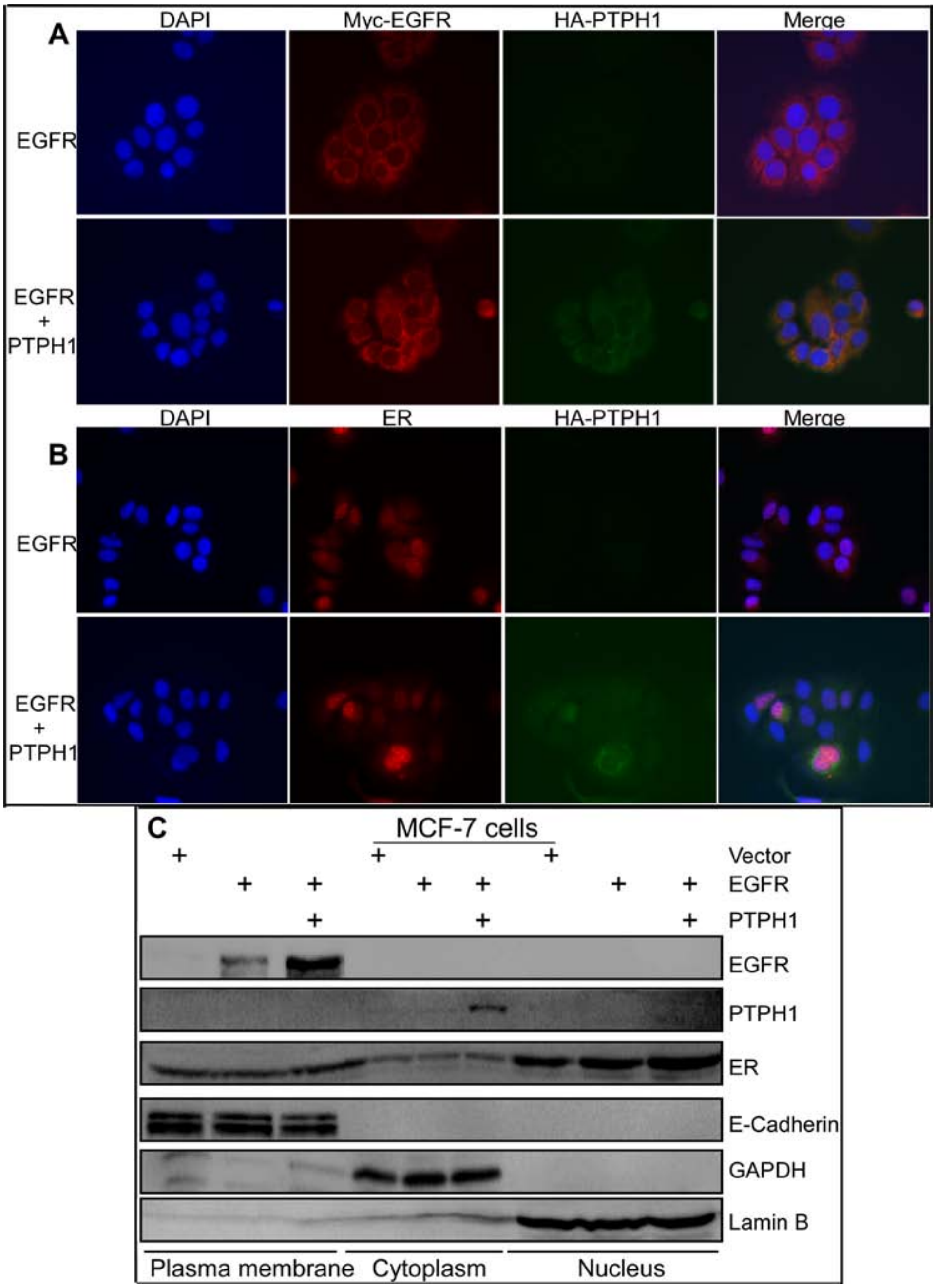

Supplementary Figure S8: PTPH expression increases the membranous EGFR and the nuclear ER accumulations. **A, B.** Myc-EGFR stably transfected MCF-7 cells with and without HA-PTPH1 co-expression were plated on cover slips and subjected to double-staining, with anti-EGFR (Rabbit, 1:100, Cy3) and anti-HA (mouse, 1:100, FITC) for **A**, and with anti-ER (rabbit, 1:100, Cy3) and anti-HA (mouse, 1:100, FITC) for **B**. Merged images were shown at right, with DAPI stained DNA at left. **C.** PTPH1 and EGFR co-expression increases the membranous EGFR and the nuclear ER localization. Cell fractionation analyses were performed as described in Figure 6C and similar results were obtained in a separate experiment.
